# Exogenous 3,3′-Diindolylmethane Improves Vanadium Stress Tolerance in *Brassica napus* Seedling Shoots by Modulating Antioxidant Enzyme Activities

**DOI:** 10.3390/biom11030436

**Published:** 2021-03-16

**Authors:** Arun Gokul, Mogamat Fahiem Carelse, Lee-Ann Niekerk, Ashwil Klein, Ndiko Ludidi, David Mendoza-Cozatl, Marshall Keyster

**Affiliations:** 1 Department of Plant Sciences, Qwaqwa Campus, University of the Free State, Phuthadithjaba 9866, South Africa; GokulA@ufs.ac.za; 2 Environmental Biotechnology Laboratory, Department of Biotechnology, University of the Western Cape, Bellville 7535, South Africa; 3341863@myuwc.ac.za (M.F.C.); 3255882@myuwc.ac.za (L.-A.N.); MendozaCozatlD@missouri.edu (D.M.-C.); 3 Plant Omics Laboratory, Department of Biotechnology, University of the Western Cape, Bellville 7535, South Africa; aklein@uwc.ac.za; 4 DST-NRF Centre of Excellence in Food Security, University of the Western Cape, Bellville 7530, South Africa; nludidi@uwc.ac.za; 5 Plant Biotechnology Research Group, Department of Biotechnology, University of the Western Cape, Bellville 7535, South Africa; 6 C.S. Bond Life Sciences Center, Division of Plant Sciences, University of Missouri, Columbia, MO 65211, USA

**Keywords:** 3,3′-diindolylmethane, antioxidant enzymes, *Brassica napus*, reactive oxygen species, vanadium

## Abstract

3,3′-diindolylmethane (DIM) belongs to a family of indole glucosinolate compounds that have been shown to improve *Brassica napus* growth through the modulation of reactive oxygen species when applied exogenously. The *B. napus* cultivar AV Garnet was previously identified as a vanadium-sensitive cultivar. Therefore, in this study we investigated whether exogenous DIM could improve the vanadium tolerance of AV Garnet. We performed the following experiments: seed germination assessment, dry weight assessment, cell viability assay, chlorophyll content assay, malondialdehyde (MDA) assay, conjugated diene (CD) content assay, hydrogen peroxide (H_2_O_2_) content assay, superoxide (O_2_^−^) content determination, methylglyoxal (MG) content determination, hydroxyl radical (**·**OH) concentration determination, ascorbate peroxidase (APX) activity assay, superoxide dismutase (SOD) activity assay, glyoxalase I (Gly I) activity assay, glutathione S-transferase (GST) activity assay and inductively coupled plasma optical emission spectroscopy (ICP-OES) analysis for vanadium content determination. Under vanadium stress, exogenous DIM increased the seed germination percentage, shoot dry weight, cell viability and chlorophyll content. Exogenous DIM also led to a decrease in MDA, CD, H_2_O_2_, O_2_^−^, MG and **·**OH, under vanadium stress in the shoots. Furthermore, DIM application led to an increase in the enzymatic activities of APX, SOD, Gly I and GST under vanadium stress. Interestingly, under vanadium stress, DIM treatment did not alter vanadium content in *B. napus* shoots. Our results indicate that exogenous application of DIM can improve *B. napus* seedling shoot growth and biomass under vanadium stress by priming the antioxidant enzymes via reactive oxygen species (ROS) signaling.

## 1. Introduction

*Brassica napus* is a plant species from the family Brassicaceae and is thought to have originated from the hybridization of two species, *Brassica rapa* and *Brassica oleracea*, between 6800 and 12,500 years ago. *B. napus* is an oil crop ranked behind soybean as the 2nd largest for oil production [1]. *B. napus* is widely used as a healthy source of vitamin E, for the production of margarine, dairy blends, animal feed, emulsifiers and cooking oils [2]. Due to its nutritional benefits in human consumption as well as animal feed, the demand for *B. napus* has rapidly increased over time [3]. However, *B. napus* yield is limited by heavy metal stress such as cadmium [4], lead [5], arsenic [6], chromium [7], mercury [8], nickel [9], copper [10] and vanadium stress [11].

Vanadium is ranked as the 5th most abundant transition metal and is 22nd among all discovered elements found in the Earth’s crust [12]. Vanadium mining occurs extensively in Australia, South Africa, China, Russia and the United States of America. Due to extensive mining, vanadium can leach into the surrounding soils and the continuous build-up can cause pollution [13]. Therefore, Vanadium is recognized as a potentially dangerous environmental pollutant in the same class as mercury, lead and arsenic [14]. Vanadium content in the Earth’s crust varies from 10 to 200 μg·g^−1^ [15] and at testing sites vanadium mostly exist as vanadium pentaoxide (V_2_O_5_), ammonium metavanadate (NH_4_VO_3_), sodium metavanadate (NaVO_3_) and sodium orthovanadate (Na_3_VO_4_) [11]. Therefore, vanadium from the soil can get into direct contact with plants and studies have shown that elevated concentrations of vanadium reduce the root and shoot growth in *Cicer arietinum* [16], *Glycine max* [17], *Citrullus lanatus* [18] and *Phaseolus vulgaris* [19]. In most cases, plants treated with excessive vanadium display chlorosis due to chlorophyll degradation [20] as well as protein degradation [21]. In addition, vanadium toxicity leads to overproduction of reactive oxygen species (ROS) (O_2_^−^, H_2_O_2_ and **·**OH), which ultimately leads to lipid peroxidation and eventually cellular death [13]. MDA and CD are oxidized by-products of membrane lipids and are considered to be bona fide indicators of lipid peroxidation in plants [22]. Heavy metals lead to the overproduction of MG, which is a reactive α, β-dicarbonyl ketoaldehyde [23]. MG at cytotoxic levels can inhibit cell proliferation, as well as causing an increased degradation of proteins through the formation of advanced glycation end products [24]. However, the production of MG under vanadium stress has not been investigated to date. To combat downstream toxicity and cellular death from reactive molecules (O_2_^−^, H_2_O_2_, **·**OH and MG), plants have developed enzymatic defense systems such as *SOD*, *APX*, *GST* and *Gly I*, which regulate (inactivate or detoxify) the reactive molecules under heavy metal stress.

The use of biomolecules to prime plant defense systems under heavy metal stresses have become an attractive strategy to prevent heavy metal-induced damage [25]. For instance, Nawaz et al. [18] used exogenous melatonin to reduce vanadium toxicity in *C. lanatus* seedlings. Parveen et al. [22] used exogenous citric acid to reduced copper stress toxicity in *Corchorus capsularis* seedlings. Bless et al. [26] used exogenous MG to alleviate zirconium toxicity in *B. rapa* seedlings. Fu et al. [27] used exogenous hydrogen sulfide to alleviate cadmium toxicity in *Hordeum vulgare*. Mahmud et al. [28] used γ-aminobutyric acid (GABA) to confer chromium stress tolerance on *Brassica juncea*. Exogenous proline and glycinebetaine were used to confer tolerance to cadmium stress on *Nicotiana tabacum* [29] and *Vigna radiata* [30]. Exogenous 5-aminolevolinic acid confers cadmium tolerance in *B. napus* [31]. Song et al. [32] used exogenous oxalic acid to improve the lead tolerance of *Larix olgensis* seedlings.

Glucosinolates are nitrogen- and sulfur-containing biomolecules, mainly found in the Brassicaceae family, with plant signaling roles under stress [33]. Glucosinolates are stable compounds under normal conditions, but they are hydrolyzed into several downstream degradation products by the enzyme myrosinase when plant tissues and cells are damaged [34]. DIM is one of the more stable by-products of indole glucosinolate breakdown and possesses signaling roles in *B. napus* [34] and *Oryza sativa* [35]. However, there are no studies that elucidate a role for DIM in heavy metal stress in plants and particularly *B. napus*. Therefore, we conducted this study to assess the effect of exogenous DIM pretreatment on *B. napus* seedlings under vanadium stress in order to elucidate potential vanadium alleviation mechanisms in plants. Our results show that DIM does protect *B. napus* against vanadium stress and our data further show that a priming of the antioxidant capacity of *B. napus* may be part of the mechanism behind the DIM-mediated alleviation mechanism against vanadium.

## 2. Materials and Methods

### 2.1. Preparation of DIM

The DIM (Sigma, St. Louis, MO, USA; ≥98% (HPLC); CAS Number 1968-05-4) stock solution was prepared as described by Gokul et al. [34].

### 2.2. Plant Growth, Treatments and Determination of Growth Parameters

Plant growth experiments were performed as per the method of Gokul et al. [34] with slight modifications. Planting (1200 seeds) was done in plastic pots (15 cm diameter), containing a nutrient rich potting mix (Stodels Nurseries, Cape Town, South Africa; 1 part Double Grow weed-free compost and 1 part Double Grow potting soil) under a 22 °C/16 °C day/night temperature cycle with a 16/8 h light/dark cycle, at a photosynthetic photon flux density of 300 μmol photons.m^−^^2^·s^−1^ (during the day phase), in a randomized design. The germination percentage of the *B. napus* seeds (Agricol, Cape Town, South Africa; AV Garnet cultivar) was determined by first pre-treating (100 mL) the pots (no seeds) with control (distilled water), 15 µM DIM, 350 µM vanadium (NaVO_3_), and 15 µM DIM + 350 µM vanadium for 24 h, followed by planting all the seeds (400 seeds per treatment) and observing the number of seeds germinating (defined as seeds with radicals 3 mm or more in length), up until no further seeds germinated. During this time, the seedlings were treated with 100 mL of control, DIM, vanadium, and DIM + vanadium. Treatments were applied twice a week for 14 days and all treatments contained 0.009% (*v*/*v*) of Tween^®^-80.

### 2.3. Biomass (Dry Weight) Assessment

At the end of the seedling stage (14 days), we removed the seedling roots to prevent erroneous data interpretation caused by possible root damage from seedling removal from the soil. Dry weight analysis was performed by drying seedling shoots (individually) per treatment at 55 °C for 48 h and the weights were subsequently recorded as described by Gokul et al. [34].

### 2.4. Cell Viability Assay Using Evans Blue Dye

Cell viability was assessed using the method of Gokul et al. [34]. Briefly, the intact seedling shoot was immersed in 0.25% (*w*/*v*) Evans blue (dye content ≥75%) and the dye uptake was measured by means of spectrophotometry at 600 nm using a FLUOstar Omega UV-visible spectrophotometer (BMG LabTech GmbH, Ortenberg, Germany).

### 2.5. Chlorophyll Content Estimation

Total chlorophyll content in the *B. napus* shoots was estimated using a method previously described by Nxele et al. [36]. Briefly, the weight of freshly harvested seedling shoots was recorded before homogenizing the shoots in 5 mL of dimethylsulfoxide (DMSO) and incubated at 65 °C for 3 h. The absorbance rates of the extract (200 μL) were recorded in a FLUOstar Omega UV-visible spectrophotometer (BMG LabTech GmbH, Ortenberg, Germany) at 645 nm and 663 nm, with DMSO used as a blank.

### 2.6. Determination of Lipid Peroxidation

Lipid peroxidation was assayed by monitoring MDA production in seedling shoots and quantified using the thiobarbituric acid reactive substances (TBARS) assay as described by Zhang et al. [37]. Briefly, shoot material (100 mg) was ground into a fine powder using liquid nitrogen. Then, the powder was homogenized in 1 mL of cold 6% (*w*/*v*) trichloroacetic acid (TCA). The supernatant (100 μL) was mixed with 400 μL of 0.5% thiobarbituric acid (TBA; prepared in 20% TCA). The resulting mixture was incubated at 95 °C for 30 min and the reaction was terminated on ice for 5 min. The mixture was centrifuged at 12,000× *g* for 5 min at 4 °C and the absorbance was recorded at 532 nm and 600 nm using a FLUOstar Omega UV-visible spectrophotometer (BMG LabTech GmbH, Ortenberg, Germany). The non-specific absorbance was subtracted and the MDA concentration was calculated using the extinction coefficient of 155 mM·cm^−1^.

### 2.7. Conjugated Diene (CD) Content Assay

A modified method by Chérif et al. [38] was followed for the CD assay. Seedling shoots from all treatments were harvested and ground into fine powders using liquid nitrogen. Then, 100 mg of fine powder was further ground in reagent 1 (3 mL of 99.9% (*v*/*v*) methanol containing 100 mM ethylenediaminetetraacetic acid (EDTA), 3 mL of 99.9% (*v*/*v*) chloroform (containing amylenes as stabilizers)), and reagent 2 (3 mL of a solution containing 5 mM EDTA and 1% (*w*/*v*) sodium chloride (NaCl)). The mixture was centrifuged at 4000× *g* for 10 min (4 °C) in glass tubes. Nitrogen gas was used to remove the chloroformic phase and the leftover residue was dissolved in 500 µL of chloroform. Nitrogen gas was used to further dry 50 µL of residue sample and the sample was dissolved in 800 µL of absolute ethanol. A fraction of the resulting supernatant (200 µL) was spectrophotometrically read at 234 nm with a FLUOstar Omega UV-visible spectrophotometer (BMG LabTech GmbH, Ortenberg, Germany) and the extinction coefficient of 26.5 mM·cm^−1^ was used to calculate the CD concentration.

### 2.8. Hydrogen Peroxide Content Determination

Hydrogen peroxide content was quantified using a H_2_O_2_ standard curve as described by Velikova et al. [39]. Briefly, shoot material (100 mg) was ground into a fine powder in liquid nitrogen. The powder was homogenized in 1 mL of cold 6% (*w*/*v*) TCA and the extracts were centrifuged at 12,000× *g* for 30 min at 4 °C. The supernatant (50 μL) was mixed with 5 mM dipotassium phosphate (K_2_HPO_4_) at pH 5.0 and 0.5 M potassium iodide (KI) in a total volume of 200 μL. The reaction mixture was incubated at 25 °C for 20 min and the absorbance readings were recorded at 390 nm using a FLUOstar Omega UV-visible spectrophotometer (BMG LabTech GmbH, Ortenberg, Germany).

### 2.9. Superoxide Content Determination

Superoxide content was quantified using the method of Gokul et al. [34]. Briefly, intact seedling shoots were submerged in a solution containing 10 mM potassium cyanide (KCN), 10 mM H_2_O_2_, 2% (*w*/*v*) sodium dodecyl sulfate (SDS), 80 mM nitroblue tetrazolium (NBT) and 50 mM potassium phosphate buffer (pH 7.0) and incubated for 20 min. After incubation, the shoots were homogenized and centrifuged at 10,000× *g* for 5 min. The supernatant was spectrophotometrically analyzed at 600 nm using a FLUOstar Omega UV-visible spectrophotometer (BMG LabTech GmbH, Ortenberg, Germany). The superoxide concentration was calculated using the NBT extinction coefficient of 12.8 mM·cm^−1^.

### 2.10. MG Content Determination

The MG content was calculated from a MG standard curve using a modified method, as described by Mostofa et al. [40]. Seedling shoots (200 mg) were homogenized in 2.5 mL 0.5 M Perchloric acid, followed by incubation on ice for 15 min. The extract was centrifuged for 10 min at 11,000× *g* at 4 °C and the supernatant (1 mL) was mixed with activated charcoal (10 mg·mL^−1^) and kept at room temperature for 15 min. The homogenate was centrifuged for at 11,000× *g* for 10 min and the supernatant was neutralized using saturated potassium hydroxide (KOH) at room temperature for 15 min. The mixture was centrifuged at 11,000× *g* for 10 min. The neutralized supernatant (650 μL) was mixed with 330 μL of 100 mM Phosphate buffer (pH 7.0) and 20 μL of freshly prepared 0.5 M N-acetyl-L-cysteine and incubated for 15 min at room temperature. The absorbance of the resulting supernatant was recorded at 288 nm with a FLUOstar Omega UV-visible spectrophotometer (BMG LabTech GmbH, Ortenberg, Germany).

### 2.11. Hydroxyl Radical Concentration Determination

The method of Halliwell et al. [41] was used to determine **·**OH concentrations in seedling shoots, with slight modifications. Intact seedling shoots were weighed and submerged in a solution containing 10 mM phosphate buffer (pH 7.4) containing 15 mM 2-Deoxy-D-Ribose. The samples were then incubated at 37 °C for 4 h. After incubation, shoots were homogenized and a volume of 0.7 mL of the homogenate was added to a reaction mixture containing 3 mL of 0.5% (*w*/*v*) TBA (made up in 5 mM sodium hydroxide (2 mL) and 1 mL of 100% (*v*/*v*) glacial acetic acid). The sample was briefly mixed by vortex and the reaction mixture was heated for 30 min at 100 °C. After the heating step, the sample was cooled on ice for 10 min. The samples were centrifuged for 5 min at 10,000× *g*, the resulting supernatant was spectrophotometrically monitored at 532 and 600 nm with a FLUOstar Omega UV-visible spectrophotometer (BMG LabTech GmbH, Ortenberg, Germany) and the ·OH concentration was determined using the extinction coefficient of 155 mM·cm^−1^.

### 2.12. Protein Extraction for Spectrophotometric Assays

Seedling shoots from all treatments were harvested and ground into a fine powder using liquid nitrogen. Shoots (200 mg) were homogenized in 1 mL of polyvinylpyrrolidone (PVP) buffer (40 mM phosphate buffer (pH 7.4), 1 mM EDTA, 5% (*m*/*v*) PVP (MW = 40,000), 5% (*v*/*v*) glycerol in distilled H_2_O) and protein concentrations were determined using the RC DC Protein Assay Kit 11 (Bio-Rad Laboratories, Hercules, CA, USA), as described by Nkomo et al. [42].

### 2.13. Ascorbate Peroxidase (APX) Activity Assay

Seedling shoot APX activities were measured in extracts using a method previously described in [43]. Briefly, the protein extracts (20 µL) were supplemented with 2 mM L-ascorbic acid and incubated for 5 min on ice. A reaction mixture of protein extract, 50 mM phosphate buffer (pH 7.0), 0.1 mM EDTA and 50 mM L-ascorbic acid was prepared. The final reaction was initiated by adding 1.2 mM H_2_O_2_ to the reaction mixture (total volume of 200 μL) and the APX activity was monitored at 290 nm using a FLUOstar Omega UV-visible spectrophotometer (BMG LabTech GmbH, Ortenberg, Germany) and calculated using the extinction coefficient of 2.8 mM·cm^−1^.

### 2.14. Total Superoxide Dismutase (SOD) Activity Assay

Seedling shoot SOD activities were measured using a method previously described in [44]. Briefly, the protein extract (10 μL) was mixed with 190 μL of the assay buffer (50 mM phosphate buffer (pH 7.8), 0.1 mM EDTA, 10 mM methionine, 5 μM riboflavin, 0.1 mM NBT) and the mixture was incubated at room temperature for 20 min under fluorescent light. The absorbance was recorded at 560 nm using a FLUOstar Omega UV-visible spectrophotometer (BMG LabTech GmbH, Ortenberg, Germany) and SOD activity was calculated based on the amount of enzyme required to cause a 50% reduction of NBT.

### 2.15. Gly I Activity Assay

The Gly I activity assay was performed according to the method of [45]. Briefly, a 180 µL reaction assay mixture (100 mM phosphate buffer (pH 7.5), 3.5 mM MG, 15 mM magnesium sulphate (MgSO_4_) and 1.7 mM glutathione (GSH)) was prepared and incubated for 10 min at room temperature. After incubation the reaction was initiated by the addition of the protein extract (20 µL) and Gly I activity was monitored spectrophotometrically at 240 nm using a FLUOstar Omega UV-visible spectrophotometer (BMG LabTech GmbH, Ortenberg, Germany).

### 2.16. Glutathione S-Transferase (GST) Activity Assay

The GST activity was measured using 1-chloro-2,4-dinitrobenzene (CDNB), as per the method of [46]. Briefly, the protein extract was mixed with 100 mM potassium phosphate buffer (pH 6.5), 1 mM GSH and 1% (*v*/*v*) absolute ethanol in a total volume of 200 µL. The reaction was initiated by the addition of 1 mM CDNB and kinetically followed by the use of a FLUOstar Omega UV-visible spectrophotometer (BMG LabTech GmbH, Ortenberg, Germany) at 340 nm. The GST activity was determined using the extinction coefficient of 9.6 mM·cm^−1^ for CDNB.

### 2.17. Inductively Coupled Plasma Optical Emission Spectroscopy (ICP-OES) Analysis

Sample digestion of stored shoot material of treated *B. napus* plants was performed according to [47]. The vanadium concentration was determined by using a Varian Vista Pro CCD simultaneous inductively coupled plasma optical emission spectrometer (ICP-OES) (Varian, Australia) using certified standards (Sigma, St. Louis, MO, USA; TraceCERT^®^).

### 2.18. Statistical Analysis

All experiments were performed six times independently. For O_2_^−^ content, cell death, **·**OH content and shoot dry weight measurements, 20 individual *B. napus* seedlings per treatment were analyzed. For all other experiments, 40 *B. napus* seedling shoots were homogenized in pools of 10 seedlings per treatment. The one-way analysis of variance (ANOVA) test was used for statistical analysis on all data, and means (for six independent experiments) were compared according to the Tukey–Kramer test at a 5% level of significance using GraphPad Prism 5.03 software.

## 3. Results

### 3.1. Exogenous DIM Improves Seed Germination under Vanadium Stress

We observed increases in *B. napus* seed germination of 108% in DIM-treated seeds and 43% in DIM + vanadium combination-treated seeds when compared to the control seeds (Table 1). A reduction in seed germination of 58% was observed when seeds were treated with vanadium (Table 1). However, seed germination in the presence of DIM + vanadium was increased by 244% compared to the vanadium-only-treated seeds (Table 1).

### 3.2. Exogenous DIM Improves Seedling Shoot Chlorophyll Content under Vanadium Stress

DIM treatment led to an increase in *B. napus* chlorophyll *a* content of 35% (Table 2). Vanadium treatment led to a reduction in chlorophyll *a* content of 21% (Table 2). However, we observed no significant differences in chlorophyll *a* content in DIM + vanadium combination-treated seedlings (Table 2). An increase in chlorophyll *a* content of 36% was observed in DIM + vanadium combination-treated seedlings when compared to the vanadium treatment (Table 2). Furthermore, we observed no significant difference in chlorophyll *b* content in DIM-treated seedlings (Table 2). However, we observed a 35% reduction in chlorophyll *b* content in vanadium-treated seedlings (Table 2). The DIM + vanadium combination treatment led to a reduction of 21% in chlorophyll *b* content (Table 2). An increase in chlorophyll *b* content of 22% was observed in DIM + vanadium combination-treated seedlings when compared to the vanadium treatments (Table 2). DIM treatment increased the total chlorophyll (*a* + *b*) content by 26% (Table 2). A reduction in total chlorophyll content of 26% was observed in vanadium-treated seedlings (Table 2). However, we observed no significant difference in total chlorophyll content in DIM + vanadium combination treatments (Table 2). An increase in total chlorophyll content of 32% was observed in DIM + vanadium combination-treated seedlings when compared to the vanadium-treated seedlings (Table 2).

### 3.3. Exogenous DIM Improves Seedling Shoot Growth and Dry Weight under Vanadium Stress

**Figure 1:**
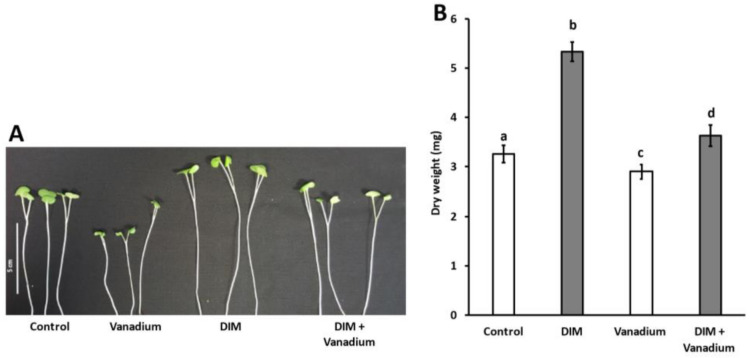
Representative visual image of *B. napus* seedling shoots (**A**) and shoot dry weight of seedlings (**B**) treated with control, DIM, vanadium and DIM + vanadium. Data represent the mean (±SE) from six independent experiments and different letters (a, b, c and d) represent statistical significance at *p* < 0.05 (Tukey–Kramer test).

An increase in growth was observed after DIM treatment when compared to the control (Figure 1A). A decrease in growth was observed in the vanadium-treated seedlings (Figure 1A). We observed similar growth for the control and DIM + vanadium combination treatment (Figure 1A). An overall increase in growth was observed in the DIM combination treatment with vanadium, when compared to the vanadium-only-treated seedlings (Figure 1A). An increase in *B. napus* dry weight of 63% was observed in DIM-treated seedling (Figure 1B). A reduction in dry weight of 38% was observed in vanadium treatment (Figure 1B). An increase in dry weight of 11% was observed in DIM + vanadium combination treatments (Figure 1B). Furthermore, an increase in dry weight of 80% was observed in DIM + vanadium combination-treated seedlings when compared to the vanadium treated seedlings (Figure 1B).

### 3.4. Exogenous DIM Reduces O_2_^−^ Content and H_2_O_2_ Content in Seedlings under Vanadium Stress

**Figure 2:**
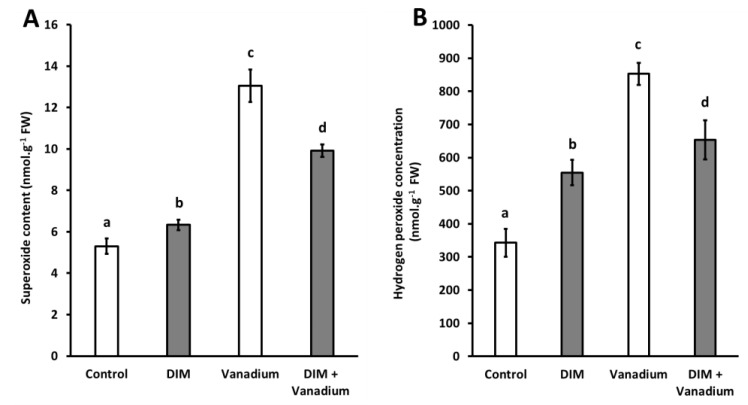
Superoxide content (**A**) and hydrogen peroxide concentration (**B**) in *B. napus* seedling shoots under control, DIM, vanadium and DIM + vanadium treatments. Data represent the mean (±SE) of six independent experiments. Different letters (a, b, c and d) represent statistical significance at *p* < 0.05 (Tukey–Kramer test).

We observed increases in O_2_^−^ content in DIM-treated seedlings (19%), vanadium treatment (146%), and DIM + vanadium combination treatment (87%), respectively (Figure 2A). However, a decrease in O_2_^−^ content of 24% was observed in DIM + vanadium combination treatment (Figure 2A). Furthermore, we observed increases in H_2_O_2_ content in DIM-treated seedlings (62%), vanadium-treated seedlings (149%), and DIM + vanadium combination-treated seedlings (91%), respectively (Figure 2B). In addition, a decrease in H_2_O_2_ content of 23% was observed in DIM + vanadium combination treatment when compared to the vanadium treatment (Figure 2B).

### 3.5. Exogenous DIM Reduces MG Content and ·OH Content in Seedlings under Vanadium Stress

**Figure 3:**
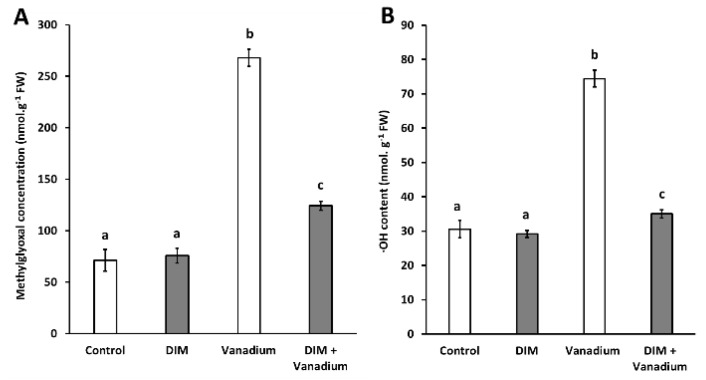
Methylglyoxal content (**A**) and hydroxyl radical content (**B**) in *B. napus* seedling shoots under control, DIM, vanadium and DIM + vanadium treatments. Data represent the mean (±SE) of six independent experiments. Different letters (a, b and c) represent statistical significance at *p* < 0.05 (Tukey–Kramer test).

We observed no significant difference in MG content in DIM-treated seedlings (Figure 3A). However, we observed increases in MG content in vanadium-treated seedlings (277%), and DIM + vanadium combination-treated seedlings (75%), respectively (Figure 3A). A decrease in MG content of 54% was observed in DIM + vanadium combination-treated seedlings when compared to the vanadium treatment (Figure 3A). Furthermore, we observed no significant difference in **·**OH content in DIM-treated seedlings (Figure 3B) but we observed increases in **·**OH content in vanadium-treated seedlings (143%), and DIM + vanadium combination-treated seedlings (15%), respectively (Figure 3B). A decrease in **·**OH content of 53% was observed in DIM + vanadium combination-treated seedlings when compared to the vanadium treatment (Figure 3B).

### 3.6. DIM Reduces MDA and CD in Seedlings under Vanadium Stress

**Figure 4:**
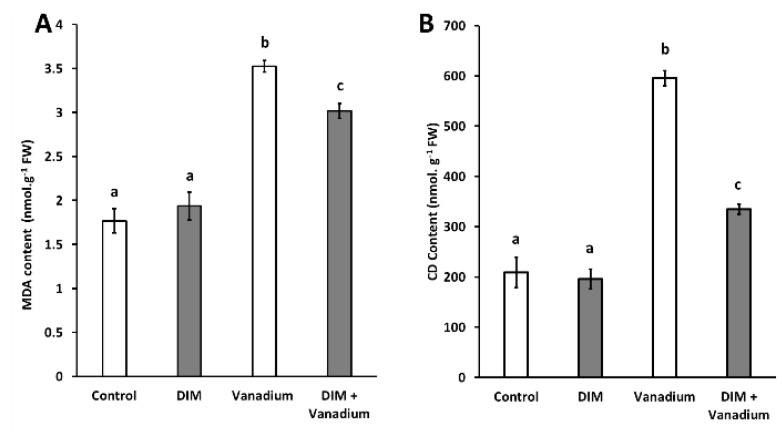
Malondialdehyde (MDA) content (**A**) and conjugated diene (CD) content (**B**) in *B. napus* seedling shoots under control, DIM, vanadium and DIM + vanadium treatments. Data represent the mean (±SE) of six independent experiments. Different letters (a, b and c) represent statistical significance at *p* < 0.05 (Tukey–Kramer test).

We observed no significant difference in MDA content in DIM-treated seedlings (Figure 4A). An increase in MDA content of 99% was observed in vanadium treatment (Figure 4A). An increase in MDA content of 71% was observed in DIM + vanadium combination treated seedlings (Figure 4A). A decrease in MDA content of 14% was observed in DIM + vanadium combination treatments when compared to the vanadium-treated seedlings (Figure 4A). Furthermore, no significant difference in CD content was observed in DIM-treated seedlings (Figure 4B). An increase in CD content of 185% was observed in the vanadium treatment (Figure 4B). An increase in CD content of 60% was observed in DIM + vanadium combination-treated seedlings (Figure 4B). Furthermore, a decrease in CD content of 44% was observed in DIM + vanadium combination treatment when compared to the vanadium-treated seedlings (Figure 4B).

### 3.7. Exogenous DIM Increases SOD Activity and APX Activity in Seedlings under Vanadium Stress

**Figure 5:**
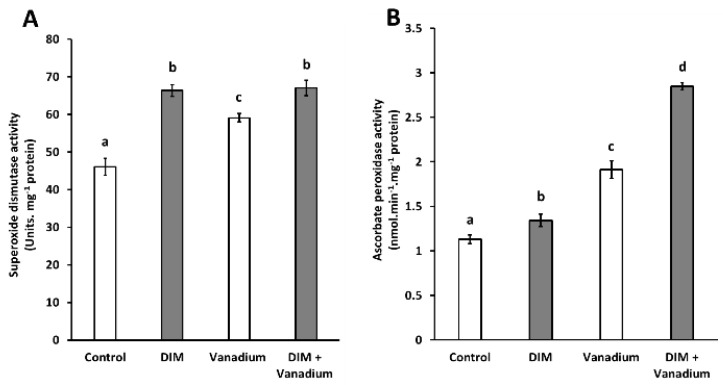
Superoxide dismutase (SOD) activity (**A**) and ascorbate peroxidase (APX) activity (**B**) in *B. napus* seedling shoots under control, DIM, vanadium and DIM + vanadium treatments. Data represent the mean (±SE) of six independent experiments. Different letters (a, b, c and d) represent statistical significance at *p* < 0.05 (Tukey–Kramer test).

We observed increases in superoxide dismutase activities in DIM-treated seedlings (44%), vanadium-treated seedlings (28%), and DIM + vanadium combination-treated seedlings (45%), respectively (Figure 5A). An increase in superoxide dismutase activity of 13% was observed in DIM + vanadium combination treatments when compared to the vanadium-treated seedlings (Figure 5A). Furthermore, we observed increases in ascorbate peroxidase activities in DIM-treated seedlings (19%), vanadium-treated seedlings (69%), and DIM + vanadium combination-treated seedlings (152%), respectively (Figure 5B). An increase in ascorbate peroxidase activity of 49% was observed in DIM + vanadium combination treatment when compared to the vanadium-treated seedlings (Figure 5B).

### 3.8. Exogenous DIM Increases Gly I Activity and GST Activity in Seedlings Shoots under Vanadium Stress

**Figure 6:**
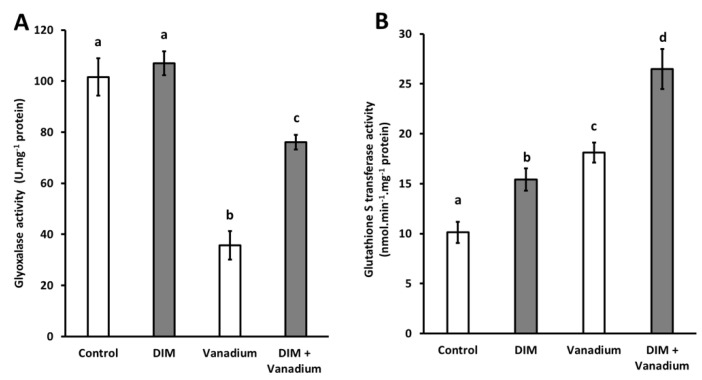
Gly I activity (**A**) and glutathione S-transferase (GST) activity (**B**) in *B. napus* seedling shoots under control, DIM, vanadium and DIM + vanadium treatments. Data represent the mean (±SE) of six independent experiments. Different letters (a, b, c and d) represent statistical significance at *p* < 0.05 (Tukey–Kramer test).

No significant difference in Gly I activity was observed in DIM-treated seedlings (Figure 6A). A decrease in Gly I activity of 65% was observed in vanadium-treated seedlings (Figure 6A). A decrease in Gly I activity of 25% was observed in DIM + vanadium combination treatment, when compared to the control (Figure 6A). Furthermore, an increase in Gly I activity of 113% was observed in DIM + vanadium combination treatment when compared to the vanadium-treated seedlings (Figure 6A). Furthermore, we observed increases in GST activities in DIM-treated seedlings (52%), vanadium treated seedlings (79%), and DIM + vanadium combination-treated seedlings (161%), respectively (Figure 6B). An increase in ascorbate peroxidase activity of 46% was observed in DIM + vanadium combination-treated seedlings when compared to the vanadium treatment (Figure 6B).

### 3.9. Exogenous DIM Reduces Seedling Shoot Cell Death under Vanadium Stress

**Figure 7:**
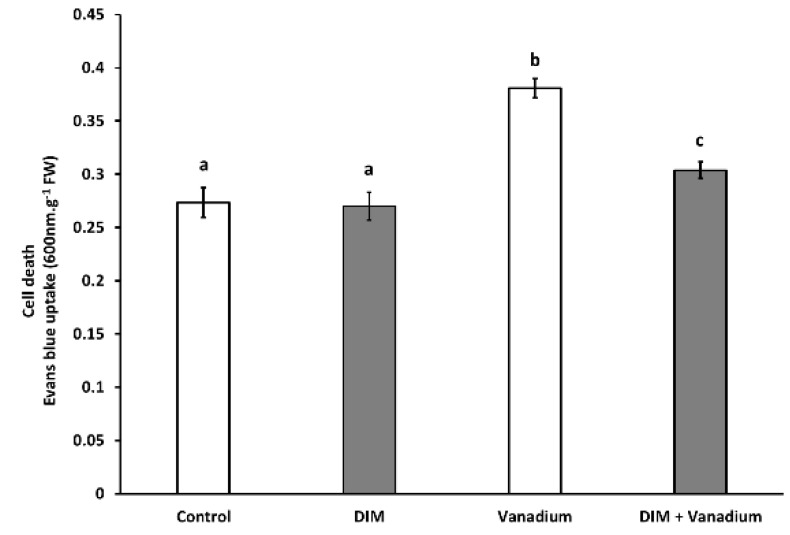
Seedling shoot Evans blue uptake (cell death) after control, DIM, vanadium and DIM + vanadium treatments. All the values are mean (±SE) from six independent experiments. Different letters (a, b and c) differ significantly at *p* < 0.05 according to the Tukey–Kramer test.

No significant difference in shoot Evans blue uptake was observed in the DIM treatments (Figure 7). An increase in Evans blue uptake of 39% was observed in vanadium-treated seedlings (Figure 7). An increase in Evans blue uptake of 11% was observed in DIM + vanadium combination treatment (Figure 7). Furthermore, a decrease in Evans blue uptake of 20% was observed in DIM + vanadium combination-treated seedlings when compared to the vanadium treatment (Figure 7).

### 3.10. Exogenous DIM Does Not Reduce Seedling Shoot Vanadium Content under Vanadium Stress

**Figure 8:**
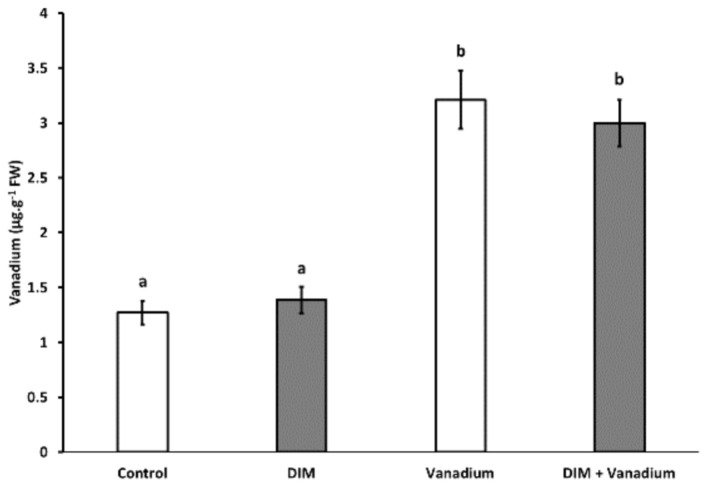
Seedling shoot vanadium content after control, DIM, vanadium and DIM + vanadium treatments. All the values are mean (±SE) from six independent experiments. Different letters (a, b) differ significantly at *p* < 0.05 according to the Tukey–Kramer test.

No significant difference in vanadium content was observed in DIM-treated seedlings when compared to the control (Figure 8). An increase in vanadium content of 153% was observed in vanadium-treated seedlings (Figure 8). An increase in vanadium content of 136% was observed in DIM + vanadium combination-treated seedlings when compared to the control (Figure 8). Furthermore, no significant difference was observed in vanadium content in DIM + vanadium combination treatment and the vanadium-only treatment (Figure 8).

## 4. Discussion

Our previous study showed that *B. napus* growth and development were affected by treatment with 350 µM of vanadium [11]. In another study, we showed that 15 µM of DIM could improve *B. napus* seedling shoot growth through the activation of ROS signaling pathways in the absence of stress [34]. Therefore, the aim of the present study was to document the effects of exogenous DIM on vanadium toxicity responses in *B. napus* using the vanadium-sensitive cultivar AV Garnet at the early seedling stage.

In this study, we observed an increase in seed germination after DIM treatment. Vanadium treatment led to a decrease in germination percentage and this result was in agreement with the observations of Wu et al. [48], in which *Medicago sativa* seed germination was decreased by vanadium treatment (50 mg·L^−1^). However, in our study, the DIM and vanadium combination treatment improved seed germination when compared to the vanadium-only-treated seeds. Furthermore, we observed taller seedling shoots after DIM treatment when compared to the control and this result agrees with our previous findings [34]. We observed stunting of seedling shoots at the harvesting stage following vanadium treatment. This result agreed with the observations of Tham et al. [49], in which seedling shoot growth was inhibited in *H. vulgare*, *Triticum aestivum*, *G. max* and *O. sativa* following vanadium treatment. In this study, however, DIM treatment showed improved seedling shoot growth even in the presence of vanadium. In addition, DIM treatment led to an increase in seedling shoot biomass (dry weight). We also observed a decrease in seedling shoot biomass in the vanadium-only-treated seedlings and this result agreed with the observations of Nawaz et al. [18], in which *C. lanatus* seedling biomass decreased in response to vanadium treatment. However, in our study, we observed an improvement in biomass in the DIM and vanadium combination treatment when compared to the vanadium-only treatment. Our observations of improved seedling shoot growth in the DIM-only and the combination treatment could be attributed to the fact that we also observed an increase in chlorophyll *a*, chlorophyll *b* and total chlorophylls in the DIM-only and the DIM and vanadium combination treatment when compared to the vanadium-only treatment. In the literature, a direct correlation between chlorophyll content and biomass exists in plants [42,50], and Nawaz et al. [18] also observed a direct correlation between biomass improvement and chlorophylls in a melatonin and vanadium combination treatment compared to vanadium-only treatment in *C. lanatus* seedlings. In addition, DIM, as a glucosinolate molecule, could also hypothetically interact directly with the chlorophyll *a* molecule and possibly lead to an increase in chlorophyll *a* content. This hypothesis is supported by Gielen et al. [51], who observed an increase in chlorophyll *a* in a *B. napus* cultivar with higher glucosinolate content.

Vanadium toxicity leads to the generation of toxic reactive molecules, which ultimately lead to plant cell death [11,52]. At moderate levels, these reactive molecules can also prime scavenging enzymes and thus the careful control of reactive compounds could be an attractive strategy to improve plant performance under vanadium stress [18]. Therefore, in this study we measured reactive oxygen species (O_2_^−^, H_2_O_2_ and **·**OH) as well as the reactive dicarbonyl compound MG. In this study, DIM treatment led to an increase in O_2_^−^ and H_2_O_2_ content and no change in **·**OH content. In addition, we observed no change in MG content after DIM treatment and this result suggests that there is no interplay between DIM and MG under non-abiotic stress (control) conditions in *B. napus*. We also observed an increase in the concentrations of O_2_^−^, H_2_O_2_, **·**OH and MG following vanadium treatment. Vanadium treatment also increased the O_2_^−^ content of AV Garnet leaves in our previous study [11], which is in agreement with the findings in the current study. An increase in H_2_O_2_ content in *C. lanatus* seedlings was also observed by Nawaz et al. [18] following vanadium treatment, which is in agreement with the findings in our study. Even though no direct link exists between **·**OH and vanadium stress in the literature, it is well known that other heavy metals trigger an increase in toxic **·**OH content in plants [53,54]. In addition, there are also no published data which suggest that vanadium stress leads to an increase in MG content in plants. However, it is well documented that other heavy metals increase MG content and this lead to cellular damage and cell death [28,55]. We also observed a decrease in the concentrations of O_2_^−^, H_2_O_2_, **·**OH and MG in the DIM and vanadium combination treatment when compared to the vanadium-only treatments. This result suggests that exogenous DIM decreased the toxicity of reactive compounds under vanadium stress, therefore leading to improved photosynthetic metabolism and ultimately an increase in biomass in *B. napus*. A similar observation was made by Mahmud et al. [28], in which the molecule GABA reduced all reactive compounds (O_2_^−^, H_2_O_2_, **·**OH and MG) through priming of scavenging enzymes to confer chromium stress tolerance in *B. juncea*.

Heavy metal toxicity, which results in an increase in reactive compounds, often leads to lipid peroxidation. Therefore, the extent of lipid peroxidation in plants can be assessed by measuring the levels of MDA and CD, respectively [56]. In our study, DIM treatment led to no change in MDA and CD content, which suggests that exogenous DIM does not lead to changes in MDA and CD content, potentially as a result of unchanged **·**OH content under DIM treatment. Furthermore, we observed an increase in both MDA and CD content in vanadium-only-treated seedlings. The result for the increased MDA content under vanadium stress is in agreement with our previous findings [11], but no direct link exists in the literature between vanadium stress and increases in CD content in plants. Nevertheless, it is observed in the literature that other heavy metals trigger an increase in CD content in plants [57]. A direct link was also observed between **·**OH content and lipid peroxidation marker content (MDA and CD) in lead-treated *Triticum aestivum* by Kaur et al. [57], which is in agreement with our observations. We also observed a decrease in both MDA and CD content in the DIM and vanadium combination treatment when compared to the vanadium-only treatments. We hypothesize that DIM lowers lipid peroxidation by decreasing **·**OH content in *B. napus* under vanadium stress. Ali et al. [58] indicated that **·**OH triggers the initiation step of the three-step lipid peroxidation reaction in plants by removing hydrogen atoms from fatty acids. Therefore, less lipid peroxidation is expected when the **·**OH content is decreased. This result was also observed by Kaur et al. [57], when sodium nitroprusside (nitric oxide donor) application reduced lipid peroxidation (MDA and CD content) by decreasing **·**OH content under lead stress.

Enzymes are required to scavenge reactive molecules and to limit cell death in plants under heavy metal stress [11,18]. Therefore, in this study we investigated the regulation of SOD (O_2_^−^ scavenger), APX (H_2_O_2_ scavenger), Gly I (MG scavenger) and GST (which scavenges organic hydroperoxides through glutathione-dependent isomerizations) under DIM, vanadium and DIM and vanadium combination treatments. DIM treatment led to an increase in SOD and APX activity. Furthermore, we observed no change in Gly I activity in the DIM-only-treated seedlings and thus we hypothesize that the unchanged MG content under DIM-only treatment led to no activation of Gly I. We also observed an increase in GST activity under DIM-only treatment when compared to the control. We postulate that the GST activity might be triggered directly by the DIM-only treatment. This hypothesis will require further investigation, although studies have shown that the glucosinolate molecule allyl isothiocyanate can increase GST activity in plants following exogenous application [59,60]. We also observed an increase in SOD and APX activity in the vanadium-treated seedlings. A SOD activity increase was also observed by Altaf et al. [61] in *O. sativa* treated with vanadium and an increase in APX activity was also observed in vanadium-treated *M. sativa* by Wu et al. [48]. The SOD activity increase in this study is also in contradiction with our results from the previous study, in which vanadium stress inhibited SOD activity in more mature AV Garnet leaves [11]. We hypothesize that the difference in results is due to growth stage differences in the two studies at the point of harvesting. In this study, we analyzed SOD activity at the seedling stage, whereas in Gokul et al. [11] we analyzed SOD activity in the leaves at the rosette stage. Our hypothesis is supported by Shah et al. [62], who observed that SOD activity decreased over time in *O. sativa* (cultivar Jaya) growth under Cadmium stress. In addition, we observed a decrease in Gly I activity following vanadium treatment. There is no published literature which shows that vanadium treatment leads to inhibition of Gly I activity in plants. However, Hasanuzzaman et al. [63] also observed a decrease in Gly I activity in *B. napus* treated with cadmium, which suggests that metals can inhibit Gly I activity. The inhibition of Gly I in our study under vanadium stress could explain the significant increase which we observed in the MG content following vanadium treatment. We also observed an increase in GST activity in plants treated with vanadium. We postulate that vanadium treatment could induce the transcription of *GST* genes, and this hypothesis is supported by findings of Lin et al. [64], in which vanadium stress induced transcription of 11 *GST* genes in *O. sativa.* In this study, we observed an increase in SOD, APX, Gly I and GST activities in the DIM and vanadium combination treatment when compared to the vanadium-only treatment. The capacity to control oxidative stress by antioxidant enzymes leads to much better plant growth under stress conditions [65]. Therefore, we suggest that the decreases observed in reactive compound contents in this study in response to the DIM and vanadium combination treatment is as a consequence of increases in SOD, APX, Gly I and GST activities in the combination treatment.

Continuous lipid peroxidation induces plasma membrane injuries under heavy metal stress, and this subsequently leads to a decrease in cell viability [66]. Therefore, to study cell viability changes in plants, Evans blue dye is used to measure membrane integrity. Plant cells with damaged membranes do not exclude the dye and therefore stain blue [67]. Therefore, in our study, we measured Evans blue uptake in the seedling shoots of *B. napus* (cultivar AV Garnet) following DIM, vanadium and DIM and vanadium combination treatments. We observed no change in Evans blue uptake in the DIM-only treatments and this finding was also observed in our previous study [11]. Furthermore, we observed an increase in Evans blue uptake in the vanadium-only treatments, and this finding was supported by the study of Imtiaz et al. [52]. We also observed a decrease in Evans blue uptake in the DIM and vanadium combination treatment when compared to the vanadium-only treatment. We postulate that DIM treatment increased SOD, APX, Gly I and GST activities under vanadium stress, which led to a decrease in toxic reactive compounds. Furthermore, the decrease in toxic reactive compounds under DIM treatment led to a decrease in lipid peroxidation, which subsequently improved the membrane integrity under vanadium stress, and this led to less uptake of Evans blue when compared to the vanadium-only-treated seedlings.

Plants possess finely tuned mechanisms which allow them to survive under heavy metal stress conditions. Plants avoid metal uptake from the surroundings or exclude it during the initial uptake process [68]. The metals which bypass the exclusion step are chelated or sequestered in order to prevent metal movement from roots into shoots [69]. In this study, we measured the vanadium content in *B. napus* seedlings shoots following treatment with DIM, vanadium and DIM and vanadium combination treatments. We observed no changes in the DIM-only treatment. This result suggests that DIM application does not change vanadium uptake from control soil environments. Furthermore, vanadium treatment led to an increase in seedling shoot vanadium content, and this result was also observed in our previous study, Gokul et al. [11], in which vanadium treatment led to an increase in vanadium content in *B. napus*. We also observed no change in vanadium content when we compared the DIM and vanadium combination treatment with the vanadium-only treatment. Because DIM treatment did not change vanadium content regulation mechanisms under normal conditions, our results suggest that there was also no regulation of vanadium uptake mechanisms under increased vanadium treatment in *B. napus* in the presence of DIM.

## 5. Conclusions

In conclusion, our results suggest that exogenous DIM treatments can enhance the vanadium stress tolerance of a vanadium-sensitive *B. napus* cultivar, AV Garnet, at the seed germination and seedling development stage. DIM treatment did not alter vanadium content in the combination treatment, which suggests that the triggered signaling events are crucial for the DIM-induced tolerance in the seedling shoots. Under vanadium stress, DIM application led to an increase in SOD, APX, Gly I and GST enzymatic activities, which led to a decrease in reactive compounds (O_2_^−^, H_2_O_2_, **·**OH and MG). The decrease in reactive compounds led to an improvement of the chlorophylls in the combination treatment. In addition, a much lower amount of reactive compounds led to a decrease in lipid peroxidation (MDA and CD content), to a decrease in cell death and ultimately to an improvement in seedling shoot growth. To our knowledge, this is the first report which indicates a positive functional role for DIM under vanadium stress in plants using pot experiments. This study highlights the potential signaling role of DIM in the regulation of ROS under vanadium stress at the early seedling development stage. A limitation of the study is that it studies only the shoot response. Therefore, future work should focus on non-soil experiments in order to analyze the root response. Further use of omics tools (transcriptomics and proteomics) could identify crucial molecular targets of DIM in *Brassica* plants and subsequently improve our understanding of DIM signaling in the near future.

## Figures and Tables

**Table 1:** *B. napus* seed germination in response to control, DIM, vanadium and DIM + vanadium treatments. Data represent the means (±SE) of six independent experiments and different letters per row indicate the mean values that are significantly different at *p* < 0.05 (Tukey–Kramer test).

	Control	DIM	Vanadium	DIM + Vanadium
**Germination %**	38.50 ± 4.50 ^a^	80.00 ± 7.00 ^b^	16.00 ± 1.50 ^c^	55.00 ± 4.00 ^d^

**Table 2:** Chlorophyll concentration in *B. napus* seedling shoots in response to control, DIM, vanadium and DIM + vanadium treatments. Data represent the means (± SE) of six independent experiments and different letters per row indicate the mean values that are significantly different at *p* < 0.05 (Tukey–Kramer test).

Chlorophyll (µg·g^−1^)	Control	DIM	Vanadium	DIM + Vanadium
*a*	143.36 ± 15.11 ^a^	194.15 ± 16.71 ^b^	112.39 ± 10.45 ^c^	152.50 ± 11.71 ^a^
*b*	58.81 ± 3.12 ^a^	61.18 ± 5,56 ^a^	38.07 ± 2.61 ^b^	46.61 ± 2.09 ^c^
*a* + *b*	202.17 ± 18.23 ^a^	255.33 ± 22.27 ^b^	150.46 ± 13.06 ^c^	199.11 ± 13.80 ^a^

## Data Availability

The data presented in this study are available in the published manuscript.
